# The role of VEGF-A_165_b in trophoblast survival

**DOI:** 10.1186/1471-2393-14-278

**Published:** 2014-08-15

**Authors:** Victoria L Bills, Maryam Hamdollah-Zadeh, Peter W Soothill, Steven J Harper, David O Bates

**Affiliations:** Microvascular Research Laboratories, School of Physiology and Pharmacology, Veterinary Sciences Building, Southwell St, Bristol, BS2 8EJ UK; Department of Obstetrics and Gynaecology, St Michael’s Hospital, Bristol, UK; Cancer Biology, Division of Oncology, University of Nottingham, Queen’s Medical Centre, Nottingham, NG2 7QT UK

**Keywords:** VEGF, Pre-eclampsia, Trophoblasts

## Abstract

**Background:**

Pre-eclampsia remains a dominant cause of maternal and fetal mortality in developed countries. In a previous prospective study we identified a fall in the VEGF-A isoform VEGF-A_165_b in the plasma of patients in the first trimester to be a predictor of later pre-eclampsia. VEGF-A_165_b has been shown to have potent cytoprotective properties in many cell types. We therefore tested the hypothesis that VEGF-A_165_b may be cytoprotective for placental trophoblasts.

**Methods:**

We used an immortalised first trimester trophoblast cell line exposed to chemical toxicity, and physiological (<2% O_2_) and atmospheric oxygen (21% O_2_) in the presence or absence of VEGF-A_165_b, angiogenic VEGF-A_165_a, a non-specific anti-VEGF-A blocking antibody (bevacizumab), or a specific anti-VEGF-A_165_b antibody. Cell viability and cytotoxicity were measured by trypan blue and LDH assay respectively.

**Results:**

Under high (21%) levels of oxygen, trophoblast viability was increased, and cytotoxicity reduced by exogenous recombinant VEGF-A_165_b (p < 0.05, n = 10) or VEGF-A_165_a. The cytoprotective effect was not seen under lower (<2%) oxygen conditions, where VEGF-A_165_b was upregulated. However inhibition of VEGF-A with blocking antibodies (bevacizumab or anti-VEGF-A_165_b) had marked cytotoxic effects under low oxygen conditions presumably through the blockade of autocrine survival pathways.

**Conclusions:**

These results show that when trophoblasts are exposed to lower oxygen tensions (as they are early in the 1^st^ trimester) endogenous VEGF-A_165_b contributes to their survival through an autocrine pathway. In contrast in high oxygen conditions exogenous VEGF-A isoforms have a greater effect on trophoblast survival.

## Background

Abnormal placental development is central to the pathogenesis of pre-eclampsia. The early placenta is made up of microvilli consisting of a mesenchymal core surrounded by cytotrophoblast stem cells that develop into either overlying syncytiotrophoblasts, or extravillous trophoblasts, which grow out from the placenta. The partial pressure of oxygen in the normal first trimester placenta has been found to be lower than 20 mmHg [1], i.e. the trophoblasts are physiologically used to a lower oxygen tension than later in pregnancy. At 10–12 weeks of gestation, trophoblast plugs loosen and oxygenated maternal blood enters the intervillous space, raising the oxygen tension [2]. This stimulates trophoblasts to differentiate into an invasive phenotype, which are less proliferative and more susceptible to apoptosis [3]. These processes are regulated by a wide variety of molecules, including hypoxia-inducible factor-1α (HIF-1α), VEGF and transforming growth factor-β [4, 5].

VEGF-A isoforms play critical roles in the development of the placenta. In the first trimester placenta VEGF-A is present in the cytotrophoblast, syncytiotrophoblast and endothelial cells in the villi [6]. Isoforms of VEGF-A exist in two distinct families, formed by alternative mRNA splicing [7]. The canonical isoforms are generated by splicing of pre-mRNA into the proximal splice site in exon 8a, after alternative splicing of exons 6 and 7, resulting in a family of isoforms with angiogenic activity, termed VEGF-A_xxx_ where xxx refers to the number of amino acids in the protein (e.g. VEGF-A_165_, VEGF-A_121_). The most widely studied isoform, VEGF-A_165_ has pro-angiogenic, pro-permeable and vasodilatory properties [8]. Alternative splicing in exon 8, 66 bases downstream in the pre-mRNA results in a family of isoforms, termed VEGF-A_xxx_b, which differ in their final six C-terminal amino acids. Thus VEGF-A_165_b, VEGF-A_121_b and VEGF-A_189_b can be generated as sister isoforms of the canonical isoforms [9]. VEGF-A_165_b and VEGF-A_121_b are anti-angiogenic in vivo. VEGF-A_165_b, the most extensively studied isoform, binds to and activates VEGF-R2, but is quantitatively and qualitatively different from VEGF-A_165_a [10], stimulating cyto-protective signalling, but not angiogenic pathways [11, 12]. In terms of cell survival therefore, VEGF-A_165_a and VEGF-A_165_b often have the same rather than contrasting properties. Although evidence is mounting for the expression and role of VEGF-A_165_a in placental tissue, little is known about the expression and function of placental VEGF-A_165_b, in particular the effect of this alternatively spliced anti-angiogenic isoform on trophoblast survival. This is in contrast to the recent demonstration of the anti-angiogenic role of VEGF-A_165_b in skin in patients with systemic sclerosis [13].

The foundations of pre-eclampsia are laid down in the first trimester, with inadequate invasion of placental trophoblasts into the maternal uterine spiral arteries. In the first trimester, women who were later to develop pre-eclampsia had lower levels of plasma VEGF-A_165_b than pregnant women who remained normotensive throughout pregnancy [14]. The finding that VEGF-A_165_b is cyto-protective for some cell types, led us to test the hypothesis that VEGF-A_165_b may contribute to trophoblast survival at a crucial time in the first trimester and deficiency of the molecule may prevent trophoblast well-being and the successful physiological process of spiral artery remodelling by trophoblast cells.

## Methods

### Cell culture

Cell viability and cytotoxicity assays were performed on HTR-8/SVneo cells, a human trophoblast cell line immortalised using SV40 (Simian Virus 40) large T antigen (Tag), a kind gift from Charles H. Graham, University of Toronto, who first established this cell line [15]. These cells can proliferate indefinitely, and have been shown to retain many of their phenotypic features of the non-transfected parent trophoblast cells such as staining for cytokeratin [16]. They are however, a transformed cell line, rather than a primary trophoblast culture.

These cells were cultured in sterile filtered RPMI 1640 media supplemented with 5% fetal bovine serum and 1% penicillin/streptomycin (RPMI/5%FBS/1%PS). Cells were incubated in atmospheric oxygen conditions (5% CO_2_ and 21% O_2_) at 37ºC in T25 flasks, starting at passage 78 and split twice weekly. HTR8/SVneo cells were cultured in RPMI/5%FBS/1%PS for at least 2 weeks to super-confluency, and formed the villous trophoblast cell columns that characterise this cell type.

### Cytoprotection assay

HTR-8/SVneo cells were plated onto 6-well cell culture plates at a density of 1x10^5^ with 2 ml RPMI/5%FBS/1%PS and incubated at 37ºC in 21% O_2_. After 24 hours the cells were rinsed in 2 ml 1xPBS and the media replaced with serum free RPMI 1640 supplemented with or without VEGF-A_165_b 40 ng/ml. After 48 hours of incubation at 37ºC media was harvested and centrifuged at 3000 rpm for 3 minutes. The resulting pellets consisted of dead HTR-8/SVneo cells, which were resuspended in 1 ml of RPMI. The remaining live cells on the surface of the wells were removed by trypsinisation (1 ml trypsin solution per well), and 2 ml RPMI added to inactivate the trypsin. Following centrifugation of the contents of the well at 3000 rpm for 3 minutes, the supernatant was removed and the pellet of live cells was resuspended in the dead cell solution to mix both live and dead cells together. The solution was pipetted thoroughly and 100 μl was transferred to an eppendorf containing 100 μl of 0.4% Trypan blue (400 mg trypan blue in 100 ml 1xPBS). Trypan blue dye is able to pass through the cell membrane of dead, but not live cells. The % viability of cells after treatment with agents was calculated as:


### Lactate Dehydrogenase (LDH) assay for cytotoxicity

The LDH assay (CytoTox 96, Promega) was carried out according to manufacturer’s instructions. Cells grown to 90-95% confluency were seeded onto 96-well sterile cell culture plates at a density of 3 × 10^4^ cells per well and 100 μl RPMI/5%FBS/1%PenStrep was added to each well before incubation at 37ºC. After 24 hours the media was removed, and wells were washed with 1 × PBS once before fresh serum free media was added (100 μl/well). The following treatments were added to the wells: 1nM or 2nM of rhVEGF-A_165_ or VEGF-A_165_b, an antibody against VEGF-A bevacizumab (25nM), or a VEGF-A_165_b antibody: clone 56–1 (R&D systems MAB3045), at 25 or 50 nM. Control wells contained serum free media only and the positive control wells contained 5 mM sodium butyrate, a known cytotoxicity inducing agent. No IgG control was included. Plates were incubated in 21%O_2_ at 37ºC. After 48 hours media (50 μl/well) was collected from wells and transferred to fresh 96-well plates for the later quantification of LDH concentration in the media after 48 hours of treatment. The original culture plates with their 50 μl/well of remaining media was then freeze-thawed at –80ºC and then 50ºC to lyse the HTR-8/SVneo cells. The resulting cell lysates were collected and transferred onto fresh 96-well plates (lysate) and both media and lysate plates were centrifuged at 1500 rpm for 10 minutes. 50 μl/well of solution in each well on both sets of plates were transferred to two new sets of plates to which the detection substrate tetrazolium salt was added for 30 minutes. Visible wavelength absorbance data were measured in a plate reader. The % cytotoxicity was calculated as:


LDH assays were repeated in triplicate and performed in physiological (<2% O_2_) as well as atmospheric (21% O_2_) conditions.

### ELISA

Cells were lysed using RIPA lysis buffer containing protease inhibitors and the protein concentration was measured using Precision Red (Cytoskeleton, Inc), according to the manufacturer’s guidelines. Pan-VEGF and VEGF_xxx_b ELISA was performed using the VEGF DuoSet ELISA kit DY293 and DY3045, respectively (R&D Systems, Abingdon, UK). This VEGF-A_xxx_b ELISA should detect all the VEGF-A_xxx_b isoforms, but not the VEGF-A_xxx_ isoforms, but the affinity of the antibodies to the ‘minor’ isoforms such as VEGF-A_121_b and VEGF-A_189_b has not been determined. Briefly, 1 μg/ml goat anti-human pan-VEGF or 2 μg/ml anti-human VEGF_xxx_b (Clone 56/1) were used as capture antibodies, overnight at room temperature (RT). After washing and blocking steps, serial dilutions of, rhVEGF_165_ or rhVEGF_165_b standards were added to each well in triplicate, at concentrations ranging from 15.625 pg/ml to 4 ng/ml. Sample lysates were typically diluted 1:5 in blocking solution and added in duplicate to each well. Either biotinylated goat anti-human VEGF (0.1 μg/ml), or mouse anti-human VEGF_xxx_b (0.25 μg/ml), was used as detection antibody. Absorbance was read in a plate reader Opsys MR 96-well plate reader (Dynex Technologies, Chantilly, VA, USA) at 450 nm, with the control reading at 570 nm. Revelation Quicklink 4.25 software was also used to construct a standard curve from mean absorbance values of standards enabling the estimation of VEGF concentration for each sample.

### Cell culture under reduced oxygen tension

The method of achieving low oxygen tension (<2%) has been described previously [17]. Cells were cultured in a hypoxia chamber (Sensotec GB300) to which a gaseous mix of 95% N_2_ and 5% CO_2_ (BOC) was connected at 20 l/min for 5 minutes and changed 12 hourly. Oxygen concentration was assessed after 12 hours using an oxygen monitor (Cambridge Sensotec GB300) and confirmed an oxygen reading of <2% O_2_.

### Statistics

Comparisons between VEGF-A_165_b treated (test) trophoblast cells were made with the serum free media (control) trophoblast cells. Statistical analyses of cell culture data were made using Unpaired t tests and one way ANOVAs, with p < 0.05 regarded as significant. Values are expressed as means ± S.E.M.

## Results

### VEGF-A_165_b is cytoprotective for trophoblasts

**Figure 1:**
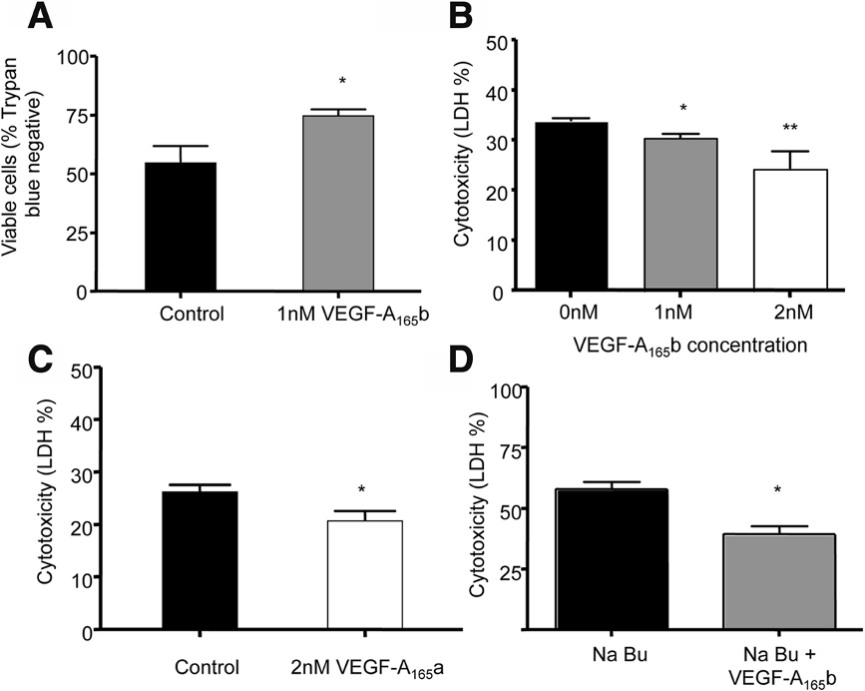
**Exogenous VEGF-A is cytoprotective for trophoblasts in atmospheric oxygen conditions. A**. HTR-8/SVneo (first trimester immortalised trophoblast) cells were cultured for 48 hours in SFM ± 1nM VEGF-A_165_b and survival assessed by Trypan exclusion. (* = p < 0.05, Unpaired t test, n = 10). **B**. Cytotoxicity assessed by LDH release (n = 19, One way ANOVA, p = 0.0019, Dunnett’s Multiple Comparison Test). **C**. Trophoblasts were cultured in SFM ± 2nM VEGF-A_165_a in 21% O_2_. After 48 hours cytotoxicity determined by LDH assay. (Unpaired t test, p < 0.05, n = 13). **D**. In the presence of 5 mM sodium butyrate (NaBu, a cytotoxic agent), addition of 1 nM VEGF-A_165_b significantly decreased the trophoblasts cytotoxicity (p < 0.05, Unpaired t test, n = 8).

To determine whether exogenous VEGF-A_165_b was cytoprotective for placental trophoblasts, 1 × 10^5^ HTR-8/SVneo trophoblast cells were cultured in serum free medium (SFM) ± 1nM VEGF-A_165_b for 48 hours in atmospheric oxygen (n = 10). Viability of cells exposed to VEGF-A_165_b, measured by trypan blue exclusion, was increased by 21% compared to control conditions (viability 75% ± 2.8 vs. 54% ± 8.1, Unpaired t test, p < 0.05, n = 10, Figure 1A). The LDH assay was then used to determine if VEGF-A_165_b increases trophoblast survival in a dose dependent manner. 19 wells were treated for 48 hours with SFM, SFM + 1nM VEGF-A_165_b or 2nM VEGF-A_165_b in 21% O_2_. A dose dependent decrease in cytotoxicity occurred (% cytotoxicity 33.6% ± 0.6, 29.2% ± 0.9, and 24.2% ± 3.5 respectively, One way ANOVA, p = 0.0019, Dunnett’s Multiple Comparison Test, Figure 1B). This was similar to that induced by VEGF-A_165_a – cytotoxicity decreased from 26.5 ± 2.4% in SFM to 20.7 ± 3.3% when cultured in 2nM VEGF-A_165_a (Unpaired t test, p < 0.05, n = 13, Figure 1C). To determine whether VEGF-A_165_b could prevent chemically induced cytotoxicity, 3 × 10^4^ trophoblast cells were cultured with 5 mM sodium butyrate ± 1nM VEGF-A_165_b. Pre-incubation of the cells with 1nM VEGF-A_165_b for two days significantly decreased Na butyrate- induced cell death (cytotoxicity 58% ± 2.7 vs. 39% ± 3.2, Unpaired t test, p < 0.05, n = 8, Figure 1D).

### Lower oxygen tensions abolish the effect of exogenous VEGF-A on trophoblast survival

**Figure 2:**
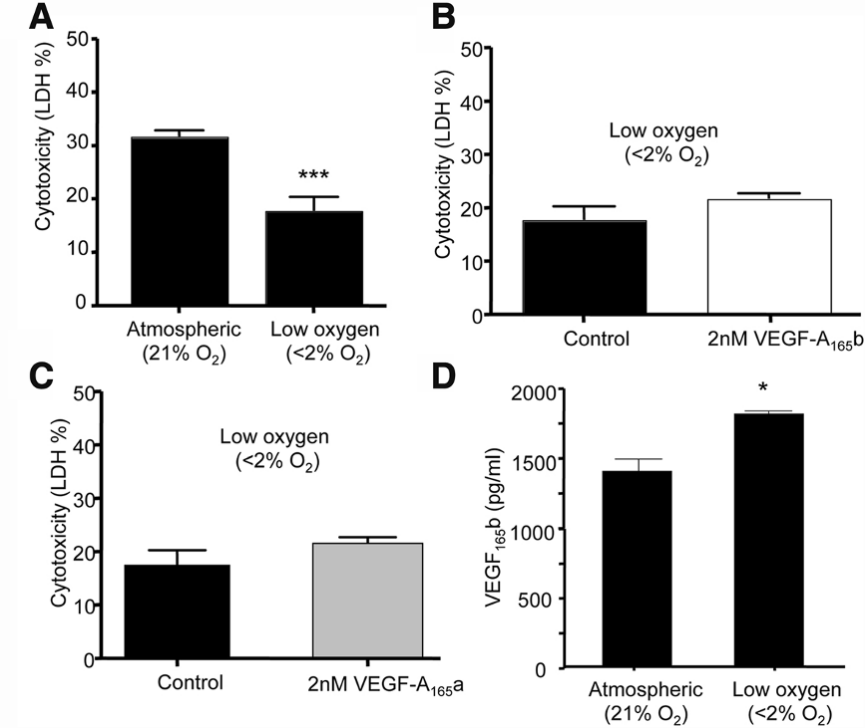
**First trimester trophoblast cells are resistant to low oxygen tensions. 1A**. Trophoblasts were cultured in 21% O_2_ or with reduced oxygen (<2%O_2_) conditions for 48 hours in serum free media (SFM) and cytotoxicity assessed by LDH release (Unpaired t test, p < 0.0001, n = 16). **B**. Trophoblasts were cultured for 48 hours in SFM or 2nM VEGF-A_165_b in <2% O_2_ and cytotoxicity assessed by LDH release (Unpaired t test p = 0.13, n = 13). **C**. Trophoblasts cytotoxicity assessed by LDH release in cells treated with 2nM VEGF-A_165_a under lower oxygen conditions. **D**. Trophoblasts were cultured for 48 hours in low or atmospheric oxygen conditions. ELISA showed that VEGF-A_165_b expression was increased by lowering pO_2_ (1812 pg/ml ±33 vs. 1407 pg/ml ±95, Unpaired t test, p = 0.016).

In the early placental environment, before 10 weeks of gestation, trophoblasts experience lower oxygen tensions of less than 20 mmHg (2.5%) [1] and VEGF-A expression has been shown to be induced by such low oxygen tensions [18]. We therefore determined whether low oxygen tension itself was cytoprotective by inducing VEGF-A expression. Immortalised HTR-8/SVneo first trimester trophoblast cells were cultured for 48 hours in serum free media (SFM) 21% O_2_ or <2% O_2_ conditions. The LDH assay was used to determine cell cytotoxicity. After 48 hours, cytotoxicity was reduced in the cells exposed to lower oxygen conditions (17.8% ± 2.6) compared to cells exposed to atmospheric oxygen (33.7% ± 0.63, n = 16, Unpaired t test, p < 0.0001, Figure 2A).

To determine whether VEGF-A_165_b could further protect cells in lower oxygen conditions, HTR-8/SVneo cells were treated with 1nM VEGF-A_165_b in SFM for 48 hours in <2% O_2_. This had no effect on cytotoxicity. To ensure that this was not due to an altered responsiveness of the cells, a separate set of cells were treated with or without 2 nM VEGF-A_165_b, which also had no effect on cytotoxicity (n = 21, Unpaired t tests, p = 0.2 Figure 2B). To determine whether VEGF-A_165_a was able to reduce cytotoxicity, cells were treated with 2nM VEGF-A_165_a in <2%O_2_. Again no reduction in cytotoxicity was seen (Figure 2C). This suggested that either the cells were no longer able to respond to VEGF-A, that they had already reached a maximal reduction in cytotoxicity, or that the cells were producing endogenous VEGF-A, and that this was acting sufficiently in an autocrine manner. We therefore measured VEGF-A_165_b levels in 1 × 10^5^ HTR8/SVneo cells incubated in either <2% O_2_ or atmospheric oxygen. After 48 hours, cells were harvested, protein extracted and expression of VEGF-A_165_b in cell lysate measured by ELISA. VEGF-A_165_b from low oxygen treated trophoblasts was significantly increased compared to cell lysate from trophoblasts cultured in 21%0_2_, when measured by ELISA (1.8 ± 0.033 ng/ml vs. 1.4 ± 0.095 ng/ml respectively, Unpaired t test, p = 0.016, Figure 2D).

### In low oxygen conditions, inhibition of total VEGF-A via Bevacizumab, or exclusively VEGF-A_165_b increases cytotoxicity

**Figure 3:**
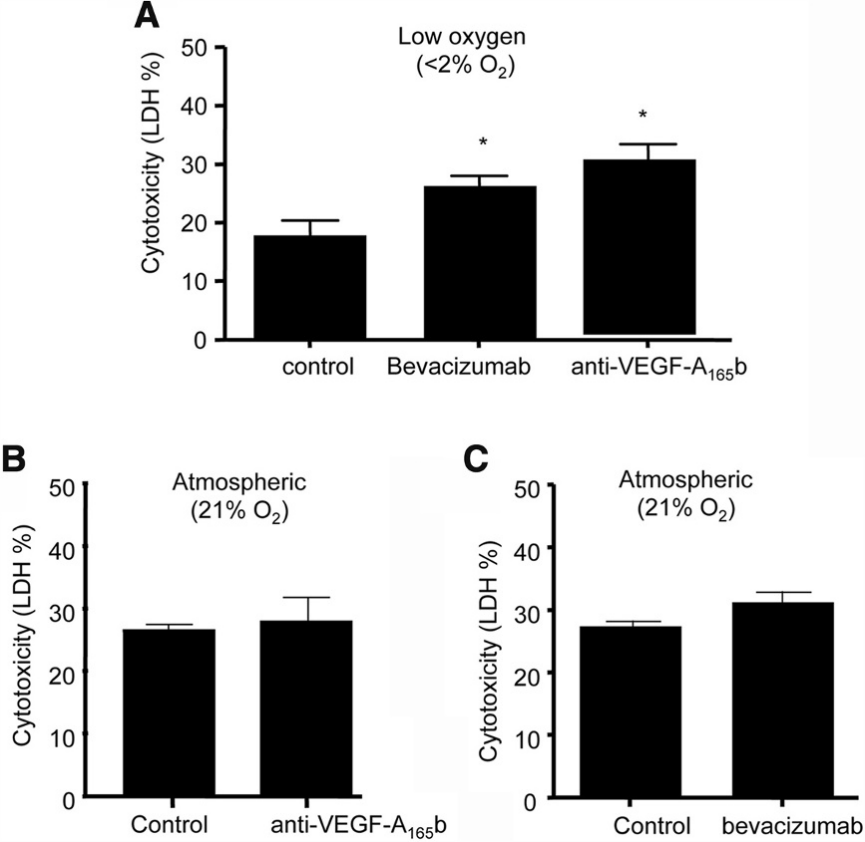
**Endogenous VEGF**_**165**_ **b is cytoprotective when pO**_**2**_ **< 2%. A**. Trophoblasts were cultured in SFM (n = 5) ± Bevacizumab 25nM (to inhibit all VEGF-A isoforms, n = 7) or 50nM anti-VEGF-A_165_b (clone 56/1, AbCam) to inhibit only VEGF-A_165_b isoforms, n = 8) for 48 hours in a hypoxia chamber (<2%O_2_) and cytotoxicity measured. (One way ANOVA, p = 0.0068, Dunnett’s Multiple Comparison Test). **B**. Trophoblasts were cultured in SFM ± 50nM anti-VEGF-A_165_b in 21% 0_2_ for 48 hours and cytotoxicity assayed. (Unpaired t test p = 0.6, n = 14). **C**. Cytotoxicity was not significantly increased in cells exposed to 25nM VEGF-A inhibitor bevacizumab (n = 23) compared with control conditions in SFM (n = 26, Unpaired T test with Welch correction, p > 0.05).

If endogenous VEGF-A is responsible for the reduction in cytotoxicity under low oxygen conditions, then inhibition of endogenous VEGF-A should be cytotoxic. In a series of 20 experiments, 3 × 10^4^ HTR-8/SVneo cells were cultured in SFM ± 25nM Bevacizumab (to inhibit all VEGF-A isoforms), or 25nM of an anti-VEGF-A_165_b monoclonal antibody (clone 56–1), to inhibit VEGF-A_165_b only, leaving VEGF-A_165_a active. Following incubation for 48 hours in a hypoxia chamber (<2% O_2_), an LDH assay was performed and cytotoxicity was expressed as relative LDH concentration in the media compared to lysate. Baseline cytotoxicity in SFM was 17.8 ± 2.6%. When cultured in Bevacizumab, cytotoxicity rose to 26.4 ± 1.6%, and was even higher when exposed to clone 56–1, 30.8 ± 2.7% (One Way ANOVA, p = 0.0068, Dunnett’s Multiple Comparison’s Test, Figure 3A). To determine whether this cytotoxicity was oxygen dependent, 3 × 10^4^ HTR-8/SVneo cells were cultured in the presence of SFM ± 25 nM Bevacizumab or 50 nM anti-VEGF-A_165_b in 21% O_2_. After 48 hours, the media and cell lysate were harvested for the quantification of LDH, and cytotoxicity was calculated. There was no significant difference in cell death following exposure to the anti-VEGF-A_165_b antibody compared to control conditions (Unpaired t Test, p = 0.6, n = 14), and a small but not statistically significant increase following exposure to Bevacizumab for 48 hours (31.4 ± 1.8% vs. 27.6 ± 0.8%, Unpaired t test, p = 0.0599, n = 49, unpaired t test with Welch correction for unequal variance, F test to compare variances p = 0.0009, Figure 3B&C).

## Discussion

We show here that trophoblasts thrive in low oxygen conditions partly by inducing VEGF-A expression resulting in an autocrine cytoprotective mechanism that is specific for the anti-angiogenic isoforms such as VEGF-A_165_b. In early placentation, extravillous trophoblasts differentiate from a proliferative to an invasive phenotype [19]. As these extravillous trophoblasts invade the maternal blood vessels they form plugs; groups of cells that occlude the spiral arteries and prevent oxygenated maternal blood from entering the intervillous space while the spiral arteries remodel to generate low resistance vessels that will provide maternal blood to the placenta [20]. Up to 10 weeks of gestation, the trophoblast cells therefore exist in a lower oxygen environment, and this results in the trophoblasts maintaining a proliferative, non-invasive phenotype [21]. Thus, the results presented here provide a potential cellular mechanism through which the trophoblasts in normal pregnancy could survive the low oxygen levels induced by their plugging of the spiral arteries.

If this upregulation of VEGF-A_165_b does not occur then the trophoblasts will suffer more cell death. In normal placental tissue, tightly regulated processes of proliferation and cell death occur continuously. Cell death may be apoptotic (programmed cell death, where organelles are packaged up to be phagocytosed), or necrotic, which involves the cytoplasm and organelles being shed into the extracellular matrix without any expenditure of cellular energy [22]. In pre-eclampsia, trophoblasts undergo increased rates of apoptosis [23, 24], and pre-eclamptic trophoblasts are more susceptible to stressful events such as TNF- α exposure, which induces apoptosis [25]. It is known that certain growth factors such as epidermal growth factor (EGF) can rescue trophoblasts from apoptosis [26]. Furthermore, in 2002 Smith *et al.* showed that insulin-like growth factor-1 (IGF-1), basic fibroblast growth factor (bFGF), and platelet derived growth factor AA (PDGF-AA) were also able to partially inhibit apoptosis induced by TNF- α and IFN-β, although VEGF-A_165_ was not able to do so [27]. The data presented here shows for the first time that the anti-angiogenic but cyto-protective isoform VEGF-A_165_b can act as a survival factor, as it rescued trophoblasts from sodium butyrate induced cell death. They also suggest that a lack of VEGF-A_165_b expression early in pregnancy, as is seen in women that go onto develop pre-eclampsia, might result in increased cell death, and hence contribute to the development of pre-eclampsia.

The expression of the pro-angiogenic factors VEGF-A and PlGF has been demonstrated in first trimester human trophoblast and placentae [17, 28]. Those authors showed that during low oxygen conditions (corresponding to before 10 weeks of gestation) the expression of VEGF-A was significantly up-regulated by 8-fold in comparison to atmospheric conditions, while PlGF expression was reduced under low oxygen tensions. However, they did not use probes or antibodies that would distinguish between the proangiogenic isoforms (VEGF-A_121_a VEGF-A_165_a, VEGF-A_189_a) or the anti-angiogenic isoforms (VEGF-A_121_b, VEGF-A_165_b, or VEGF-A_189_b).

The mechanism of action of VEGF-A_165_b on cytoprotection is still not yet clear. The expression of all three VEGF-A receptors (VEGFR1 or Flt-1, VEGFR2 or KDR, and VEGFR3) has been demonstrated in trophoblast cells [28, 29]. VEGF-A_165_a exerts its effects through VEGFR-2, whereas VEGF-A_165_b has been shown to act by preventing VEGF-A_165_a acting on VEGFR2 and by acting directly on VEGFR1 in podocyte epithelial cells and endothelial cells. Recently, VEGF-A_165_b has been shown to act as a cytoprotective agent on retinal pigmented epithelial cells and neurons through VEGFR2 but its mechanism of action on trophoblast survival is not yet known.

This work shows that VEGF-A_165_b addition to cultured trophoblasts in high oxygen conditions reduces cytotoxicity, and although addition of VEGF-A_165_b to cells under low oxygen conditions does not increase survival, specific inhibition of the VEGF-A_165_b isoform increases trophoblast death, suggesting that VEGF-A_165_b is a trophoblast survival factor both when administered exogenously in conditions of high pO_2_, and via an autocrine pathway during low pO_2_. The measured increase in VEGF-A_165_b during low pO_2_ was relatively small (30%), but it is difficult to extrapolate from this to the local concentration at the cell membrane. This work also shows that low pO_2_ increases the expression of VEGF-A_165_b by trophoblast cells in culture, suggesting that exogenous VEGF-A_165_b does not reduce cell death under low pO_2_ because endogenous VEGF-A_165_b, present in abundance, is already fulfilling the survival role. However, because the anti-VEGF-A_165_b antibody inhibits endogenous VEGF-A_165_b, a resulting increase in trophoblast cytotoxicity was observed. It is therefore likely that under low pO_2_ conditions VEGF-A_165_b isoforms play the more important role in trophoblast survival, and the finding that low pO_2_ stimulates the expression of VEGF-A_165_b supports this hypothesis. However, although total VEGF-A inhibition and specific inhibition of VEGF-A_165_b had similar effects, this does not rule out an overlapping role for VEGF-A_165_a. In addition, this work demonstrates reduced trophoblast death in low pO_2_, and increased cytotoxicity with VEGF-A_165_b inhibition. Therefore, the reduction of VEGF-A_165_b at 12 weeks of gestation seen in the plasma of women who will later develop pre-eclampsia may be reflecting an (as yet) unidentified pathological process in the trophoblasts, which prevents trophoblasts from producing sufficient VEGF-A_165_b, which if secreted in adequate amounts would help to promote trophoblast survival, and these increased levels would be reflected in the plasma. Thus under normal pregnancy conditions, the trophoblast survival in the uterine spiral artery plugs enables the appropriate remodelling of the artery to a low resistance vessel capable of providing sufficient maternal blood to the placenta. If the trophoblasts do not produce sufficient VEGF-A_165_b then they would undergo apoptosis in the low oxygen conditions of the first trimester, and the plug would be unsustainable, and hence not allow the arteries to undergo appropriate remodelling.

The above hypothesis makes a number of assumptions that should be made clear. First, the oxygen tension in the trophoblasts in vivo rises from 18 mmHg (2.5%) at 8 weeks to 40 mmHg (5%) at 10 weeks, and then to 90-100 mmHg (11-13%) at later time points. Thus 21% oxygen is hyperoxic for trophoblasts, in vivo. It will therefore be important to determine in a more physiologically relevant model (such as isolated perfused placenta), the effects of increasing oxygen tension on cytotrophoblast survival. Secondly, the effects of low oxygen tension are likely to be complex at tensions lower than 2%. It is possible that very low oxygen levels (e.g. <0.1%) may induce a true “hypoxia” in trophoblasts, and the effect of this on VEGF splicing has not been determined. We were unable to regulate the oxygen levels below 2% due to the sensitivity of the sensor, so it is possible that lower oxygen tensions were seen. Finally, trophoblasts grown in culture are unlikely to behave as they do in vivo, and so the extrapolation of these data to human physiology must by definition be speculative, but these findings provide a rationale for a more in depth investigation using intact placental tissues. Moreover, further work is now necessary to determine whether VEGF-A_165_b is able to induce the invasive properties of trophoblast cells. In addition, the LDH assay does not differentiate between necrosis and apoptosis. Future experiments using more specific methods such as flow cytometry are required, as this would allow the effect of VEGF-A_165_b on the entire trophoblast cell cycle to be determined. Moreover, we did not compare bevacizumab or anti-VEGF-A_165_b antibodies to a control IgG, as the former is a human and the latter a mouse antibody. Previous studies have shown that treatment of trophoblasts with IgG by itself has no effect on trophoblast cell death [30], but further experiments should include this as a control. Finally, these experiments have not yet clarified the relative functional effects of the pro- and anti-angiogenic VEGF-A isoforms on trophoblast proliferation and invasion, including which isoform is the more important depending on the oxygen tension of the environment. Co-culture experiments with decidua and trophoblast, at differing oxygen tensions and exposure to different doses of the two VEGF-A isoforms are now required, as it is not yet possible to make a leap from effects of exogenous and endogenous VEGF in culture to the effects of VEGF isoforms in humans.

## Conclusion

These experiments have shown that low pO_2_ increases the production of VEGF-A_165_b by placental trophoblasts, and that blocking VEGF-A_165_b under these conditions leads to trophoblast cell death. Therefore VEGF-A_165_b may be a survival factor for trophoblasts during the first trimester of pregnancy, such that VEGF-A_165_b deficiency may decrease the ability of trophoblasts to survive and subsequently invade the maternal spiral arteries during the physiological process of spiral artery remodelling. It is well established that inadequate spiral artery remodelling is associated with the development of pre-eclampsia later in the pregnancy. These molecular findings of reduced VEGF-A_165_b being associated with trophoblast toxicity in the (physiologically) low oxygen levels of first trimester of pregnancy positively corresponds to the previously reported finding that women who eventually develop pre-eclampsia have reduced plasma levels of VEGF-A_165_b during their first trimesters. These findings may imply a time and anatomical specific VEGF-A_165_b deficiency in the pathophysiology of pre-eclampsia.

## Abbreviations

ANOVA: Analysis of variance
bFGF: Basic fibroblast growth factor
BOC: British Oxygen Company
EGF: Epidermal growth factor
ELISA: Enzyme linked immunosorbent assay
FBS: Fetal bovine serum
Flt-1: fms-like tyrosine kinase
HIF: Hypoxia inducible factor
IFN: Interferon
IGF: Insulin like growth factor
IgG: Immunoglobulin G
KDR: Kinase insert domain receptor
LDH: Lactate dehydrogenase
mRNA: messenger ribonucleic acid
PBS: Phosphate buffered saline
PDGF-AA: Platelet derived growth factor
PlGF: Placental growth factor
pO2: Partial pressure of oxygen
PS: Penicilli/Streptomycin
RIPA: Radioimmunoprecipitation assay
RPMI: Roswell Park Memorial Institute
SEM: Standard error of the mean
SFM: Serum free medium
TNF: Tumour necrosis factor
VEGFR: Vascular endothelial growth factor receptor.
